# Charge carrier mobility in hybrid halide perovskites

**DOI:** 10.1038/srep12746

**Published:** 2015-08-03

**Authors:** Carlo Motta, Fedwa El-Mellouhi, Stefano Sanvito

**Affiliations:** 1 School of Physics, AMBER and CRANN Institute, Trinity College, Dublin 2, Ireland; 2 Qatar Environment and Energy Research Institute, Doha, Qatar

## Abstract

The charge transport properties of hybrid halide perovskites are investigated with a combination of density functional theory including van der Waals interaction and the Boltzmann theory for diffusive transport in the relaxation time approximation. We find the mobility of electrons to be in the range 5–10 cm^2^V^−1^s^−1^ and that for holes within 1–5 cm^2^V^−1^s^−1^, where the variations depend on the crystal structure investigated and the level of doping. Such results, in good agreement with recent experiments, set the relaxation time to about 1 ps, which is the time-scale for the molecular rotation at room temperature. For the room temperature tetragonal phase we explore two possible orientations of the organic cations and find that the mobility has a significant asymmetry depending on the direction of the current with respect to the molecular axis. This is due mostly to the way the PbI_3_ octahedral symmetry is broken. Interestingly we find that substituting I with Cl has minor effects on the mobilities. Our analysis suggests that the carrier mobility is probably not a key factor in determining the high solar-harvesting efficiency of this class of materials.

Hybrid halide perovskites have made a breakthrough in the field of organic solar cells^1^. Their unique energy-harvesting efficiency, combined with the low manufacturing costs position them as an ideal materials class to focus research. The conversion of sunlight into electrical power has recently surpassed the outstanding efficiency of 15% for both mesoporous metal-oxide scaffolds and in planar heterojunction architectures^2^,^3^,^4^ and the latest studies report a value exceeding 20%^5^. A unique property of these materials is the ability to both act as light-harvesting medium and as charge carrier transporter. However, despite the intense research carried out in the past two years, some questions remain open regarding the nature of the material’s working principles. For instance, it is still under debate whether the photo-generated charges have an excitonic or a free-carrier character, with results pointing towards contrasting conclusions^6^,^7^,^8^. Also, the origin of the high efficiencies of perovskite-based solar cell devices still needs to be unraveled, and it is not completely clear how the mobility of the active layer influences the overall performance.

Savenije *et al*.^8^ have recently performed microwave photo-conductance and photo-luminescence experiments, measuring a mobility of 6.2 cm^2^/Vs at 300 K. They found a band-like dependence of the mobility with temperature with a slope of *T*^−1.6^. At the same time, using transient THz spectroscopy, Wehrenfennig and coworkers have shown that CH_3_NH_3_PbI_3_ exhibits long charge-carrier diffusion lengths, exceeding 1 *μ*m, and a high-frequency mobility of 8 cm^2^/Vs, an indeed remarkable result for a solution-processed material^7^. They inferred that the low bimolecular recombination rate arises from a spatial separation of electrons and holes in the system. Finally, early dielectric measurements^9^ suggest a picosecond relaxation process at room temperature.

To our knowledge, to date no theoretical calculations have addressed the problem of evaluating the conductivity and charge mobility in hybrid perovskites. Motivated by this gap in the knowledge and by the intriguing rôle that the charge transport is expected to play, here we present a first-principles analysis of the transport properties of organolead halide perovskites. In particular we consider the methyl-ammonium lead-iodide perovskite, CH_3_NH_3_PBI_3_, which is the archetype of this class of materials. The cubic, tetragonal, and orthorhombic phases are explored. We show that, depending on the phase of the material and the doping level, the mobility spans a range between 5 and 12 cm^2^/Vs for holes and 2.5 and 10 cm^2^/Vs for electrons. Furthermore, our results suggest that Cl doping has little impact on the transport properties.

The electronic structure of CH_3_NH_3_PBI_3_ has been calculated with the all-electron fhi-aims code at the level of the generalised gradient approximation (GGA) in the Perdew-Burke-Ernzerhof (PBE)^10^ parameterization. Long-range van der Waals interactions are included via the Tkatchenko and Scheffler (TS) scheme^11^, which is constructed over a GGA and a pairwise dispersive potential. In order to verify the goodness of the bandstructure, additional calculations with the HSE^12^ and HSE06^13^ functional have been performed. Since these give us rather similar mobilities than those obtained with GGA, the results are not presented here. The reciprocal space integration was performed over an 8 × 8 × 8 Monkhorst-Pack grid^14^ in the case of the cubic cell, and a 6 × 6 × 4 one for both the tetragonal and the orthorhombic. A pre-constructed high-accuracy all-electron basis set of numerical atomic orbitals was employed, as provided by the fhi-aims “tight” default option. Structural optimization was performed with the Broyden-Fletcher-Goldfarb-Shanno algorithm^15^, with the crystal geometry determined by optimizing both the internal coordinates and the supercell lattice vectors with a tolerance of 10^−3^ eV/Å and with the constraint of orthogonal cell vectors.

The charge mobility has been determined by mean of the semiclassical Boltzmann theory within the constant relaxation time approximation, as implemented in the BoltzTrap code^16^. The code has been interfaced with fhi-aims and uses the fhi-aims-calculated wave-functions and eigenvalues. A very dense *k*-point sampling of 32 × 32 × 32 (32768 *k*-points over the full Brillouin zone) has been employed for the cubic cell, while for the tetragonal and orthorhombic cells it was reduced to 18 × 18 × 12.

We now briefly summarize the key steps of the scheme. From the first-principles bandstructure, *ε*_*i*,**k**_, the *α* component of the group velocity for a charge carrier in the *i*-th band is obtained as


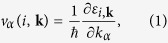


and used to compute the conductivity tensor


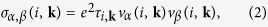


where *e* is the electronic charge and *τ*_*i*,**k**_ is the relaxation time. By integrating *σ*_*α*,*β*_(*i*, **k**), one can extract the conductivity as a function of the temperature, *T*, and the chemical potential, *ν*,


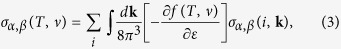


where *f* is the Fermi-Dirac distribution function. Note that *ν* is determined by the number of free carriers or, equivalently, by their concentration. Once the conductivity is known, the charge mobility reads


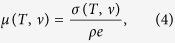


where *ρ* is the free carrier (electron or hole) concentration. While Eq. (3) is in principle exact within the semiclassical theory, it is very hard to compute unless some assumptions are taken. Therefore, for practical purposes the transport properties are calculated by introducing two approximations: (i) the relaxation time *τ* is constant, i.e. it is independent of the temperature, the band index and the chemical potential; (ii) the band structure is treated as independent of temperature or doping and, therefore, is fixed independently of the chemical potential. Nonetheless, despite such approximations, the method has been successful to predict the transport and thermoelectric properties of many materials^17^,^18^,^19^,^20^.

Here we will mainly focus on the tetragonal phase, the room temperature one, but results will be presented also for the cubic and orthorhombic. The geometries for the three phases are optimised by initializing the structures according to the experimental diffraction data^21^. In order to determine the impact of the molecular orientation on the stabilization of the inorganic matrix, which we previously discussed in the case of the cubic cell^22^, we consider two initial orientations for the CH_3_NH_3_ molecule, namely along (001) and (110). The relaxed structures are shown in Fig. 1 and will be referred to as *tetra1* and *tetra2*, respectively. The configurational energy minimum is strongly correlated to the orientation of the organic cations, in fact by only twisting the methyl-ammonium orientation two qualitatively different configurations emerge. Indeed, our calculations confirm the strong mutual connection between the organic and inorganic subsystems. As a result of the interaction between the N and the I species, the (001) orientation induces an alternate tilting of the PbI_3_ octahedra of ~14° with respect to the (001) axis [Fig. 1(b)]. In contrast, the (110) orientation induces an alternate tilting of the same amount, ~14°, in the (110) plane [Fig. 1(c)], while no disorder occurs along the (001) axis. The lattice parameter of *tetra1* and *tetra2* are 9.01 Å 8.72 Å 12.36 Å and 8.71 Å 8.71 Å 12.78 Å respectively, in agreement with experiments^21^. The energy of such two configurations is also similar, differing by 20 meV.

The electronic band structures of the two tetragonal phases look also very similar, as depicted in Fig. 2. We verified that the bottom of the conduction band mainly originates from the *p* orbitals of Pb, while the top of the valence band is derived from the *p* orbitals of I. The HOMO and LUMO of methyl-ammonium are located approximately at ±5 eV below the valence band maximum (VBM). The major difference in the bandstructure consists in a reduction of the gap from 1.7 eV to 1.5 eV in the case of *tetra2*, arising from a downshift of the Pb-5*p* band at the conduction band edge, with a larger separation from the manyfold composed of a mixture of Pb-*p* and I-*p*, *d* bands. Accordingly, the band curvature increases around Γ. A larger conductance close to the conduction band edge is thus expected for *tetra2*.

The diagonal components of the conductivity tensor, *σ*_*α*_ = *σ*_*α*,*α*_, for the three phases of CH_3_NH_3_PbI_3_ are reported in Fig. 3 as a function of the chemical potential. In the cubic phase the metal sublattice is almost isotropic along the three unit cell dimensions. Thus, although the molecular cations may have different orientations, we find that the conductivity itself is isotropic. In fact, the bands around the Fermi level, *E*_F_, originate only from Pb and I. In contrast, due to symmetry lowering the conductivity of the tetragonal and orthorhombic structures displays a significant anisotropy. For both *tetra1* and *tetra2*, *σ*_*x*_ and *σ*_*y*_ assume very similar values. Interestingly, in *tetra1 σ*_*x*,*y*_ is larger than *σ*_*z*_, while for *tetra2* we see the opposite behaviour. This can be explained in terms of the distortion of the octahedra. In *tetra1*, the octahedra tilting occurs along the *z* axis, therefore the overlap of the I-*p* orbital reduces and the transport along that direction is suppressed. In contrast, the disorder in *tetra2* is mainly in the *x*, *y* plane, while along *z* the I-Pb-I bonds are aligned causing *σ*_*z*_ to be larger. For the orthorhombic geometry, being it qualitatively similar to *tetra2*, the same considerations can be made. We point out here that our calculations are performed without the inclusion of spin-orbit coupling (SOC). Indeed, SOC is known to play a role in lead-iodide materials, mainly due to the presence of Pb. However, after comparing the bandstructures with those obtained with SOC, we noticed that the major effect is a renormalization of the energy gap. For completeness, we computed the effective masses along two high-symmetry directions around the Γ point with and without the SOC, as reported in Table 1. In agreement with previous calculations^23^,^24^, it turns out that the effect of SOC is sizeable, yet not dramatic.

We can now turn our discussion to the mobility, which has been calculated from Eq. (4) by considering *τ* = 1 ps, as determined experimentally in Ref. 9. The agreement that we find between our calculated mobilities and those recently measured experimentally suggests that such estimate is indeed a valid one. The specially averaged holes and electrons mobilities, *μ*, are plotted in Fig. 4 as a function of the charge carrier concentration, *ρ*, a quantity that is experimentally hard to determine with certainty, as it depends on the device processing, and on the presence of defects and impurities. It has been often argued that in most of the measurements *ρ* is actually very low, corresponding to a nearly intrinsic semiconductor^21^. In any case, our calculations show a generally weak dependence of the mobility on carrier concentration for both electrons and holes. In particular we predict mobilities ranging from 5 to 12 cm^2^/Vs for holes and from 2.5 to 10 cm^2^/Vs for electrons. These values are in excellent agreement with the most recent measurements^7^.

When one compares the different phases it appears clear that the cubic structure presents the highest mobility for holes and for electrons in the high doping regime. Then for holes the mobility of all the other phases is essentially the same, while for electrons there is more spread with the *tetra2* and *tetra1* displaying the highest and lowest values, respectively. This essentially follows the size of the relative effective masses, as shown in Table 1. It is however important to remark that at room temperature the molecules in the tetragonal phase are almost free to rotate, so that one expects the actual measured mobility to compare with an average of those of *tetra1* and *tetra2*.

In light of our results, we suggest that the spectacular light-harvesting performances of perovskite solar cells should not be ascribed to large charge carrier mobilities, as previously speculated^21^. Although the values that we have found are to be considered large for solution processed materials, they are not substantial in absolute terms, in particular if compared to those of conventional semiconductors like Si or Ge, which are three orders of magnitude larger. As such, the exceptionally long carrier lifetime of hybrid perovskites should be considered as the principal source of their high efficiencies.

A still outstanding issue is whether or not mixing Cl and I (Cl doping) has an impact on the charge transport of hybrid perovskites. Indeed, it has been argued that Cl doping would improve the charge transport within the perovskite layer^25^, giving rise to better performance in solar cell devices. Other works have shown that mixed halide perovskites have a bimolecular recombination rate an order of magnitude lower than that of their tri-iodide counterpart^7^. In order to partially solve this issue we have calculated the average mobilities of CH_3_NH_3_PbI_3−*x*_Cl_*x*_ for *x* = 1, 2 [see lower panel of Fig. 4]. The calculations have been performed here for the tetragonal structure. Our results show that the mobility assumes values comprised between 2–5 cm^2^/Vs, i.e. it lies within the same order of magnitude than CH_3_NH_3_PbI_3_, regardless of the Cl doping level. Clearly our results represent only an upper bound in this case, since impurity scattering may lower down the relaxation time and hence the mobility. In fact, we neglect here the effect arising from the spontaneous disorder of alloys, therefore we consider only the crystalline component of the mobility. Nevertheless, our estimate suggests that the improvement in the device efficiency has a different origin, and we endorse the idea that Cl doping does not have much detrimental effects on the transport but may help the crystalline growth of the material.

Finally, we consider an additional aspect. It is understood that dynamic effects play a key role in materials containing organic and inorganic sublattices. As mentioned before, at room temperature the organic cations are free to rotate, and recent studies suggest that the typical time scale for the CH_3_NH_3_ rotation is of the order of 1 ps^26^. We are thus interested in determining the impact of the lattice dynamics on the transport properties of this class of materials. To this end, we have performed Born-Oppenheimer molecular dynamics (MD) at 300 K with a cubic 2 × 2 × 2 supercell of CH_3_NH_3_PbI_3_. The temperature is kept constant by the use of the Bussi-Donadio-Parrinello thermostat^27^. The system is thermalized with a 5 ps run, and the dynamics of the following 1 ps is subsequently analyzed. In this time interval, the electronic bandgap is found to have an average value of 1.75 eV and a mean square deviation of 80 meV. We have sampled 20 equally spaced frames of the last 1 ps, and computed the Boltzmann transport conductivity (*σ*/*τ*) as previously for the selected configurations. Figure 5 displays the heat plot of the conductivity as a function of the chemical potential *ν* and the time during the last ps of the MD trajectory. By inspecting the trajectory, we can also confirm that the cation orientation fluctuates, i.e. that the MA molecule rotates within that time interval. This is supported by the variation of the total cation polarization, which is depicted with triangles in the figure. Such quantity is bound between 0 and 1, and clearly spans almost all the range within 1 ps. The bandgap variations, due to the molecular rotation, are clearly observed. However, apart from this minor effect, the conductivity remains independent of the thermal configurational fluctuations, especially close to the band edges. More noticeable differences appear at ~2 eV above the CBM, which however is not relevant to transport in realistic conditions. This analysis confirm that our results are robust against dynamical effects caused by thermal fluctuations.

As a final consideration, we want to emphasize that our analysis can be taken as a tool for extracting the relaxation time. Indeed the good agreement between our calculations and the most recent experiments sets the relaxation time in the ps range. This is the typical time scale of the CH_3_NH_3_ rotation at room temperature. This suggests that the mobility is probably limited by phonon scattering, in particular from those soft vibrations involving the interplay between the organic/inorganic sublattices, which is responsible for the carrier relaxation. This work motivates further study on the theoretical determination of *τ* accounting for the electron-phonon scattering and for the other sources of scattering like impurities and defects.

In summary we have evaluated the charge carrier mobilities of hybrid Pb-halide perovskites, by using a combination of rigorous density functional theory and semiclassical Boltzmann transport in the constant relaxation time approximation. Our results show that the calculated mobilities are in the experimental range, once the relaxation time is taken to be 1 ps. The particular crystal structure and the possibility of Cl doping do not affect the above results significantly.

## Additional Information

**How to cite this article**: Motta, C. *et al*. Charge carrier mobility in hybrid halide perovskites. *Sci. Rep*. **5**, 12746; doi: 10.1038/srep12746 (2015).

## Figures and Tables

**Figure 1:**
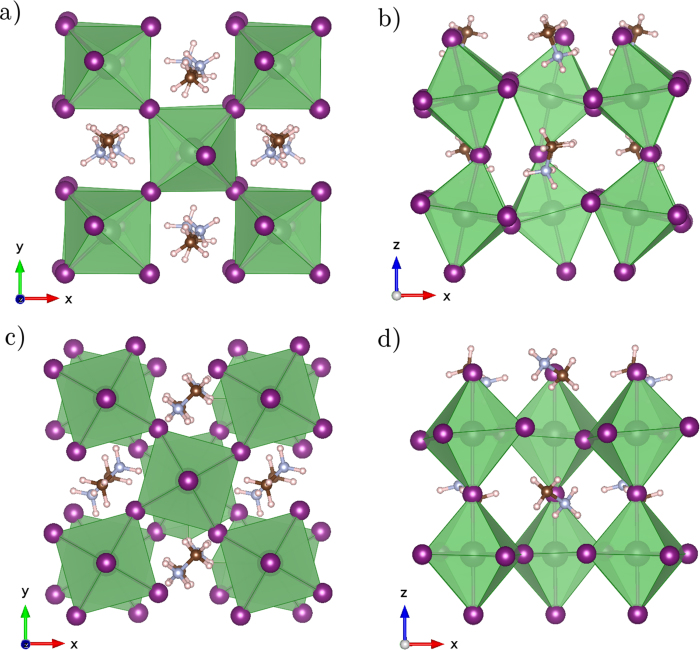
Optimized structures for the tetragonal phase of CH_3_NH_3_PbI_3_. Top and side view of *tetra1* are in (**a**) and (**b**) and for *tetra2* in (**c**) and (**d**). The two structures are obtained by initializing the crystal relaxation with the cation oriented along (001) and (110), respectively.

**Figure 2:**
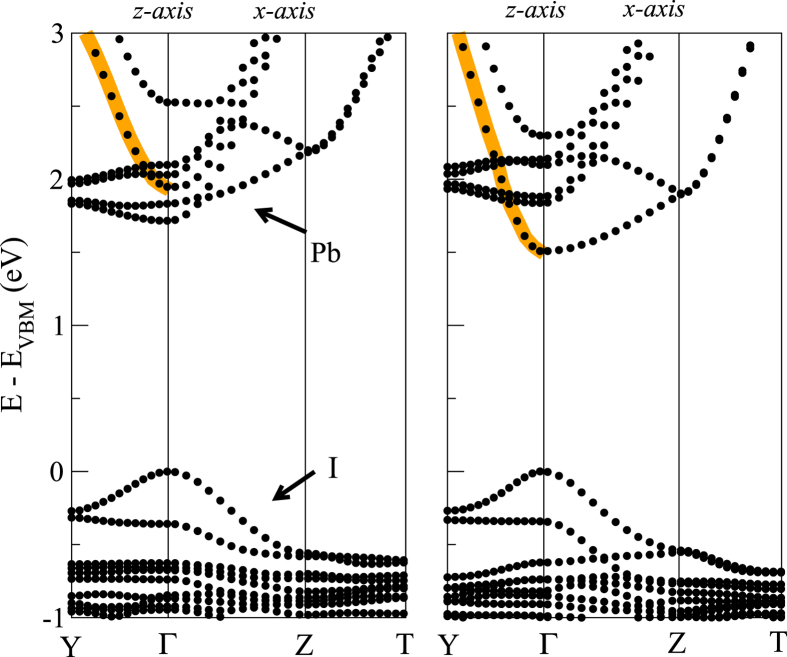
Electronic properties of the tetragonal phase of CH_3_NH_3_PbI_3_ in the *tetra1* (left) and *tetra2* (right) configurations.

**Figure 3:**
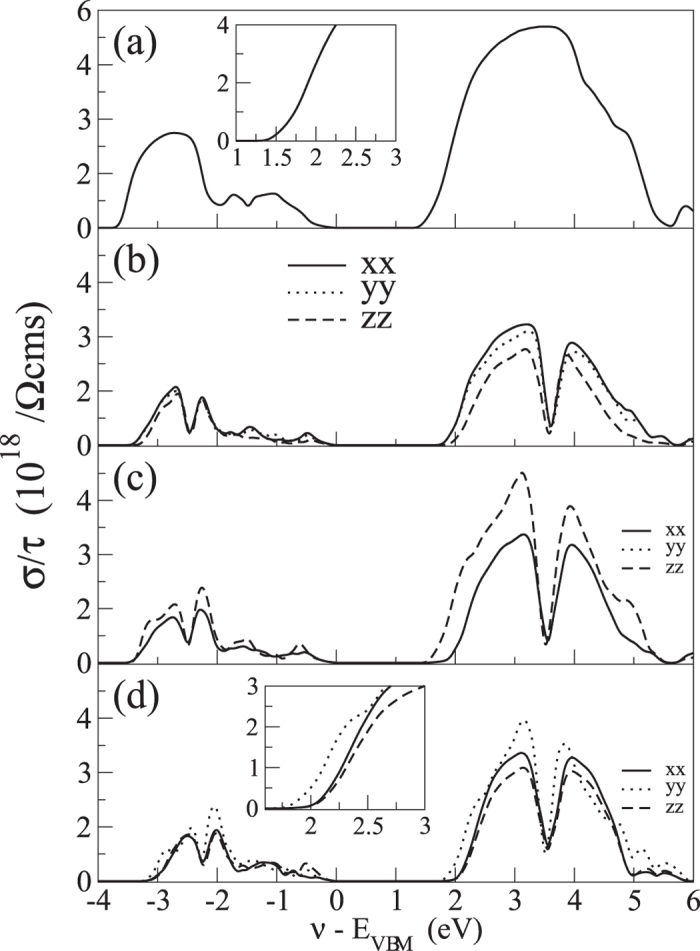
Conductivity, *σ*/*τ*, as a function of the chemical potential for the cubic (**a**) *tetra1* (**b**) *tetra2* (**c**) and orthorhombic (**d**) phases of CH_3_NH_3_PbI_3_. The solid, dotted and dashed lines represent the conductivity tensors *σ*_*xx*_, *σ*_*yy*_ and *σ*_*zz*_.

**Figure 4:**
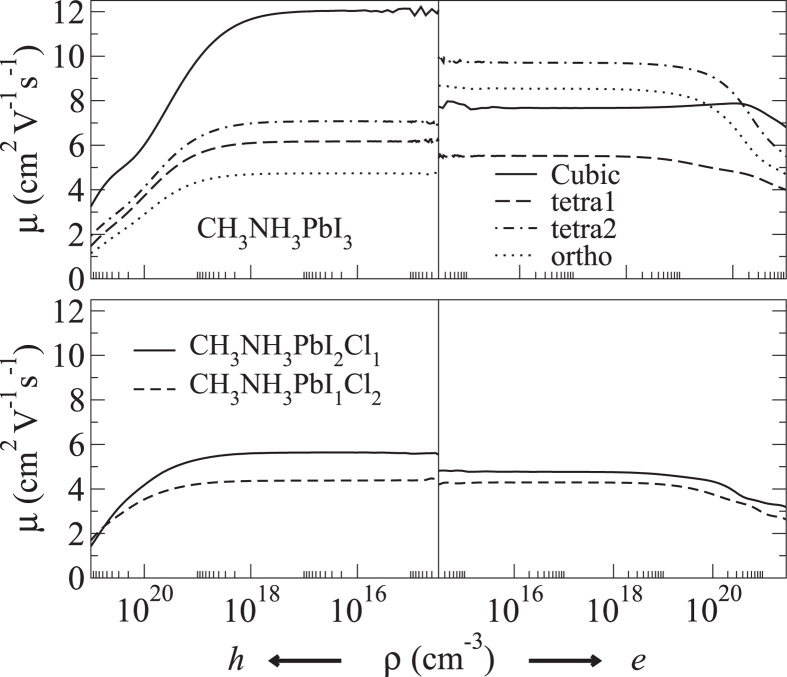
Average hole and electron mobility for the different phases of CH_3_NH_3_PbI_3_ (upper panel) as a function of the charge concentration, *ρ*. The average is taken over the three diagonal components of the mobility tensor. In the lower panel the same quantity is plotted for Cl-doped perovskites.

**Figure 5:**
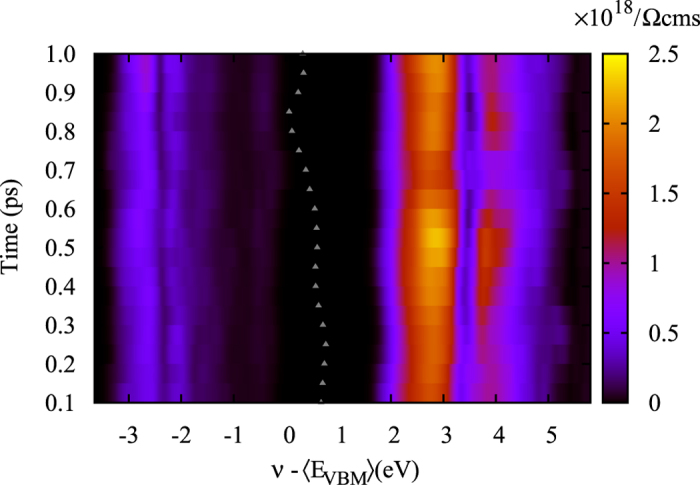
Heat map of the charge carrier conductivity *σ*/*τ* of CH_3_NH_3_PbI_3_ as a function of the chemical potential (*v*) and the MD time elapsed after an equilibration phase of 5 ps. The grey triangles represent the total polarization of the molecules contained in the supercell.

**Table 1:** Effective masses calculated with and without SOC for holes (m_*h*_) and electrons (m_*e*_) calculated by parabolic fitting along the directions Γ(0,0,0) → R (0,0,0.5) and Γ → Z (0.5,0.5,0).

	tetra1		tetra2	
	m_*h*_	m_*e*_	m_*h*_	m_*e*_
	w/o SOC			
Γ → R	0.12	0.50	0.09	0.03
Γ → Z	0.11	0.12	0.11	0.36
	SOC			
Γ → R	0.19	0.21	0.10	0.07
Γ → Z	0.13	0.10	0.15	0.13

Values are given relative to the electron mass.
